# Recruitment Variability of Coral Reef Sessile Communities of the Far North Great Barrier Reef

**DOI:** 10.1371/journal.pone.0153184

**Published:** 2016-04-06

**Authors:** Heidi M. Luter, Alan R. Duckworth, Carsten W. Wolff, Elizabeth Evans-Illidge, Steve Whalan

**Affiliations:** 1 NAMRA and the Australian Institute of Marine Science, Arafura Timor Research Facility, Darwin, NT, Australia; 2 Australian Institute of Marine Science, Townsville, QLD, Australia; 3 Central Caribbean Marine Institute, Little Cayman, Cayman Islands; 4 Marine Ecology Research Centre, School of Environment, Science and Engineering, Southern Cross University, Lismore, NSW, Australia; Leibniz Center for Tropical Marine Ecology, GERMANY

## Abstract

One of the key components in assessing marine sessile organism demography is determining recruitment patterns to benthic habitats. An analysis of serially deployed recruitment tiles across depth (6 and 12 m), seasons (summer and winter) and space (meters to kilometres) was used to quantify recruitment assemblage structure (abundance and percent cover) of corals, sponges, ascidians, algae and other sessile organisms from the northern sector of the Great Barrier Reef (GBR). Polychaetes were most abundant on recruitment titles, reaching almost 50% of total recruitment, yet covered <5% of each tile. In contrast, mean abundances of sponges, ascidians, algae, and bryozoans combined was generally less than 20% of total recruitment, with percentage cover ranging between 15–30% per tile. Coral recruitment was very low, with <1 recruit per tile identified. A hierarchal analysis of variation over a range of spatial and temporal scales showed significant spatio-temporal variation in recruitment patterns, but the highest variability occurred at the lowest spatial scale examined (1 m—among tiles). Temporal variability in recruitment of both numbers of taxa and percentage cover was also evident across both summer and winter. Recruitment across depth varied for some taxonomic groups like algae, sponges and ascidians, with greatest differences in summer. This study presents some of the first data on benthic recruitment within the northern GBR and provides a greater understanding of population ecology for coral reefs.

## Introduction

Coral reefs exhibit remarkable biodiversity [1]. Although the conspicuous scleractinian corals form key structural components of coral reefs numerous other groups play important functional roles. Notably, reef-consolidating algae [2] and sponges play vital roles in nutrient cycling and aid in benthic-pelagic energy coupling [3]. The underlying resilience of coral reefs, in part, relies on the maintenance and persistence of these coral reef communities through space and time [4], particularly for sessile benthic taxa with dispersive larval or propagule phases.

Knowledge of recruitment, larval dispersal and population connectivity of benthic sessile invertebrates is critical to the management and conservation of coral reefs [5–8]. Population connectivity of marine sessile invertebrates has largely been determined from population genetics [5], which often depicts complicated patterns of larval dispersal. Larval dispersal of coral reef invertebrates is often characterised by endogenous recruitment, but with enough long-distance dispersal to provide variable levels of population subdivision for scleractinian corals [9,10], octocorals [11] and sponges [12,13]. While assessments of larval dispersal are important to establish levels of population maintenance, collecting data on spatio-temporal variability in larval recruitment is also important [8].

Determining the spatial scales of community recruitment contributes to our understanding of resilience, maintenance and persistence of coral reefs, however, there is a large focus on documenting recruitment dynamics of scleractinian corals (e.g. [14–17]). The dedicated effort to understanding population demographics of scleractinian corals has resulted in valuable knowledge aiding how we manage these ecosystems, particularly when data show patterns of reef degradation [18–20].

Scleractinian coral recruitment studies have relied on a combination of the use of recruitment tiles (e.g. [21]) and field surveys (e.g. [15]); while broad scale interpretations of coral recruit variability are difficult, the resilience of reefs is strongly linked to recruitment potential [22,23]. Moreover, the potential for shifting taxonomic states in coral reefs following disturbance, such as coral-algae phase shifts [24], highlights the importance of understanding the dynamics and scales of recruitment variability [22]. Coral recruitment can vary greatly across many spatial scales, including between coral reefs [15,25,26], among reefs patches with reef systems [27], within reef patches [28] and between experimental recruitment tiles [21,29,30]. Recruitment variability also occurs among depths [16] and over time [15,27,31]. Interpreting drivers that contribute to coral recruitment variability is complex, but can include both abiotic (e.g. light intensity and water flow) and biotic (e.g. competition and predation) influences [15,31], as well as spatio-temporal environmental stochasticity [32]. In contrast to the many published studies examining coral recruitment, there are few studies that have investigated recruitment patterns of other sessile organisms, such as sponges, bivalves and ascidians, on coral reefs [33]. Often this recruitment data for non-scleractinian organisms is incidental to more focused coral recruitment studies (e.g. [27,29]). As such, our overall knowledge of non- scleractinian coral reef invertebrates is poorly developed, thereby hindering a broader understanding of community coral reef recruitment.

The broad objective of this study was to begin to meet some of those knowledge gaps of recruitment patterns of benthic coral reef communities (i.e. scleractinian and non-scleractinian coral reef taxa) within a region of Torres Strait, northern Australia. Torres Strait forms the northern most region of the Great Barrier Reef (GBR). While there is limited peer reviewed data on distribution and abundance of sessile coral reef taxa in Torres Strait (e.g. [34,35]), information of non-scleractinian coral reef recruitment studies are, to our knowledge, non-existent in central Torres Strait. Therefore, the specific aim of this study was to examine and quantify recruitment assemblage structure of sessile organisms across a range of spatial and temporal scales, to establish spatio-temporal variability between and within coral reefs in central Torres Strait.

## Materials and Methods

### Study site and plate deployment

The study was conducted at Masig and Marsden Islands in central Torres Strait, Australia (S1A Fig). Both islands consist of sand cays with fringing coral reefs, with a reef profile typically comprising a slope descending at an angle ranging from 20–60° from 6 m, terminating at a sand bottom at 15 m. To examine differences in recruitment patterns of sessile invertebrates at a range of spatial scales relevant to the two islands, the design of the study allowed us to examine variation in recruitment patterns at spatial scales from 5 km (between islands), 200 m (between locations), 20 m (between sites), depth (6 m vs. 12 m) and 1 m between tiles (S1B Fig). Settlement plates were deployed at each of the three locations on the northern side of each island. Each location was further divided into three sites, with each site having two depth categories: shallow (6 m) and deep (12 m). Five settlement plates, placed 1 m apart, were deployed at each site x depth combination, using the direct attachment method of [21]. Briefly, 11x11 cm terracotta tiles with pitted surfaces were anchored 1 cm above the reef to provide settlement surfaces on both sides of each plate.

Assessment of temporal patterns was made possible by deploying seasonal sets of plates at the start of the Australasian summer (November) and winter (May). Plates were deployed for six months to allow comparisons over summer and winter over a two-year period (November 2006 to May 2008). At the end of each season, the top and underside of each plate were photographed *in situ* and a new plate was deployed. Representative sponge specimens were removed from tiles during the winter 2007 sampling and preserved in 70% ethanol to facilitate higher taxonomic identifications.

The study area lies within Australian jurisdiction of the Torres Strait Protected Zone, where marine resource management is undertaken by the Australian Fisheries Management Authority (AFMA) under the Torres Strait Fisheries Act 1984. AFMA officers were consulted prior to the commencement of this study, and confirmed that the deployment of settlement of tiles required for this study was not a matter for their regulation, and did not require a permit under their act. The study area also lies within the traditional lands and seas of Torres Strait traditional owners. Their consent to the study was obtained via a consultative process coordinated by the Reef and Rainforest Research Centre, which administered all Torres Strait research conducted through the Marine and Tropical Science Research Facility (MTSRF), which funded the study. This study did not involve endangered or protected species.

### Photographic analysis

An underwater close-up frame, adapted to accommodate either an Olympus C-7070 or Canon IXUS 850IS camera in underwater housings, was constructed to photograph settlement tiles at a fixed distance and to record site and tile information. Both cameras have identical lenses and sensor-resolution; hence images produced are comparable in quality and view. The recruitment of sessile invertebrates was determined for both abundance and percent cover. To determine the abundance of each taxon, an overhead transparency marked with a square was overlaid on a PC-screen. All images of tiles were displayed by Microsoft Windows XP “Picture and Fax ViewerTM” and enlarged by clicking the zoom-in button sufficient times to identify each organism. To measure the surface area occupied by each taxon, a 40-point grid was overlaid on the PC-screen image. When analysing images for both abundance and percent cover, the square grid was reduced by a 1 cm margin to eliminate any potential edge effects. Identification to species or genus level could not be established for many of the recruits due to their small size, which is not uncommon in recruitment studies [36,37]. Therefore, recruit assemblages were categorized into broad taxonomic groups (e.g. sponges, ascidians, bryozoans, corals, polychaetes, bivalves, algae and diatoms). In addition, sponges were identified to species level if possible.

### Data analysis

All statistical analyses were performed at the taxon level (e.g. sponge, algae, polychaeta), with further analysis for sponge species that were positively identified. Multivariate permutational analysis (PERMANOVA) was used to examine differences in invertebrate recruitment patterns over various spatial scales using a balanced 5-factor nested design. Factors in the model were Season (fixed), Island (fixed), Location (random, nested within Island), Site (random, nested within Location) and Depth (random, nested within Site) and permutations were based on the Bray-Curtis resemblance matrix generated from log (x+1) transformed data. The unconstrained principal coordinate analysis (PCoA) was used to visually compare recruitment patterns of sessile invertebrates from both islands. Individual PERMANOVA tests (9999 permutations) based on the Euclidean distance matrix were performed to examine variability in recruit abundance, assemblage structure, and the individual taxa between all spatial scales at each season separately. For the individual tests, differences were considered significant at a lower p-value of <0.01 to reduce the risk of a Type 1 error. The nested design used in this study allowed for (pseudo) variance components to be compared between spatial scales and seasons [38,39]. All analyses were performed using PRIMER 6/PERMANOVA+ v1.0.2 (Plymouth, UK). Initial analyses revealed no significant difference in invertebrate recruitment patters (e.g. recruit abundance and percent cover) between the two years; therefore, only data from the second year (e.g. May 2007 to May 2008) is presented.

## Results

**Fig 1:**
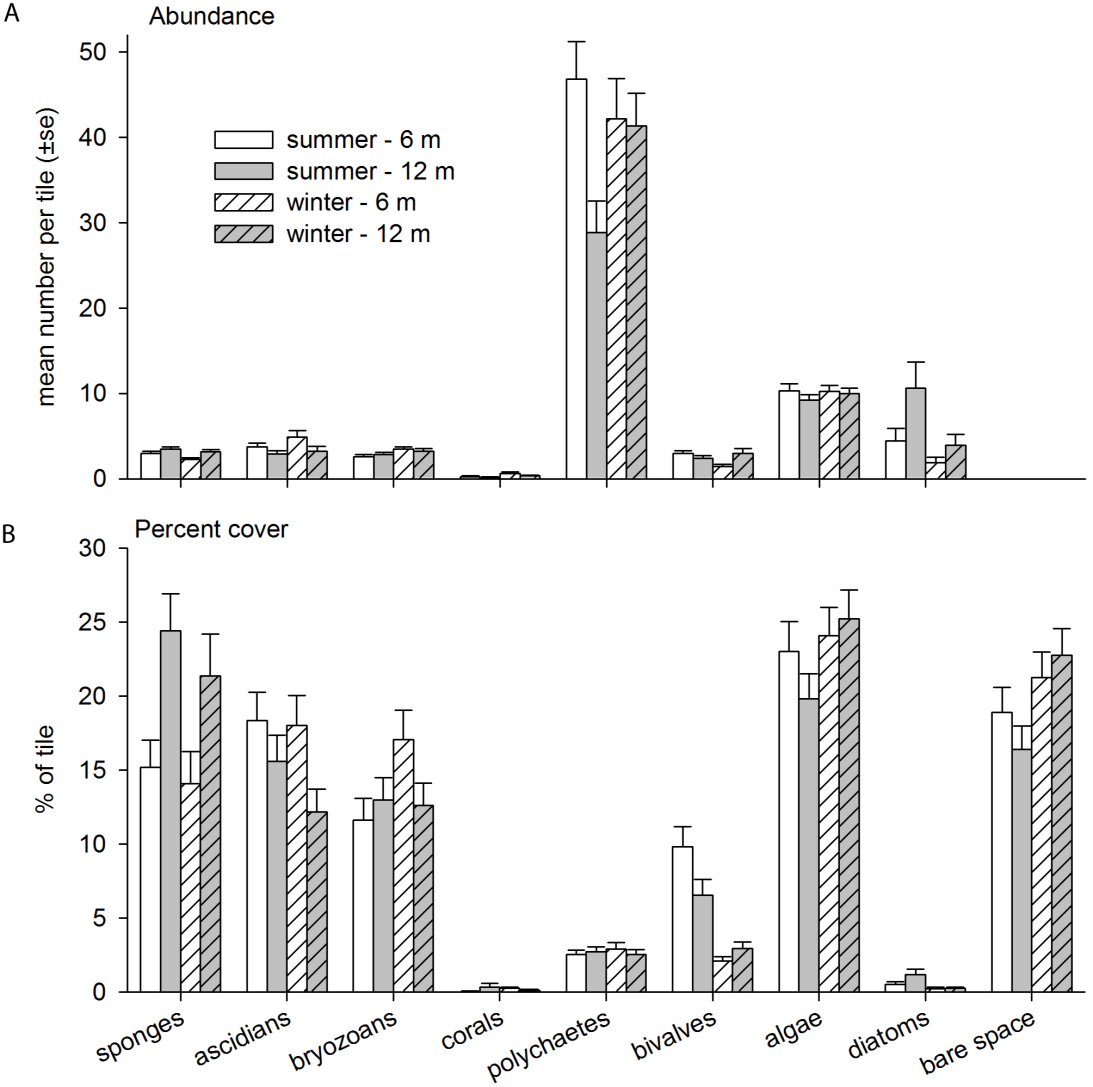
Mean abundance (A) and percent cover (B) of each of the taxa for the year 2 sampling period. Both seasons and depths depicted.

While both the top and underside of the settlement plates were photographed, >90% of the top side was bare space with very low recruitment of unidentified algae; no other organisms recruited to the top of the plate. Due to the very low recruitment on the top side of tiles, only data from the undersides are presented. In total, eight broad taxonomic groups recruited to the tiles over the course of the two-year study, including sponges, ascidians, scleractinian corals, bryozoans, polychaetes, bivalves, algae and diatoms (Fig 1; S2 Fig). Polychaetes were the most numerically dominant taxa observed, with average numeric recruitment abundances being four times higher than any other taxa (Fig 1A). Scleractinian corals displayed the lowest recruitment with an average of ≤ 1 recruit per tile observed (Fig 1A). Although polychaetes were the most numerically abundant taxa, they occupied a very low percentage cover (means ± 1 S.E., 3.0 ± 0.6%) of the settlement tiles (Fig 1B). On the other hand, groups with more encrusting prostrate morphologies such as algae (22.0 ± 1.1%), sponges (16.7 ± 1.4%) and ascidians (16.4 ± 1.4%) comprised a greater percentage of the tile surface (Fig 1B).

**Fig 2:**
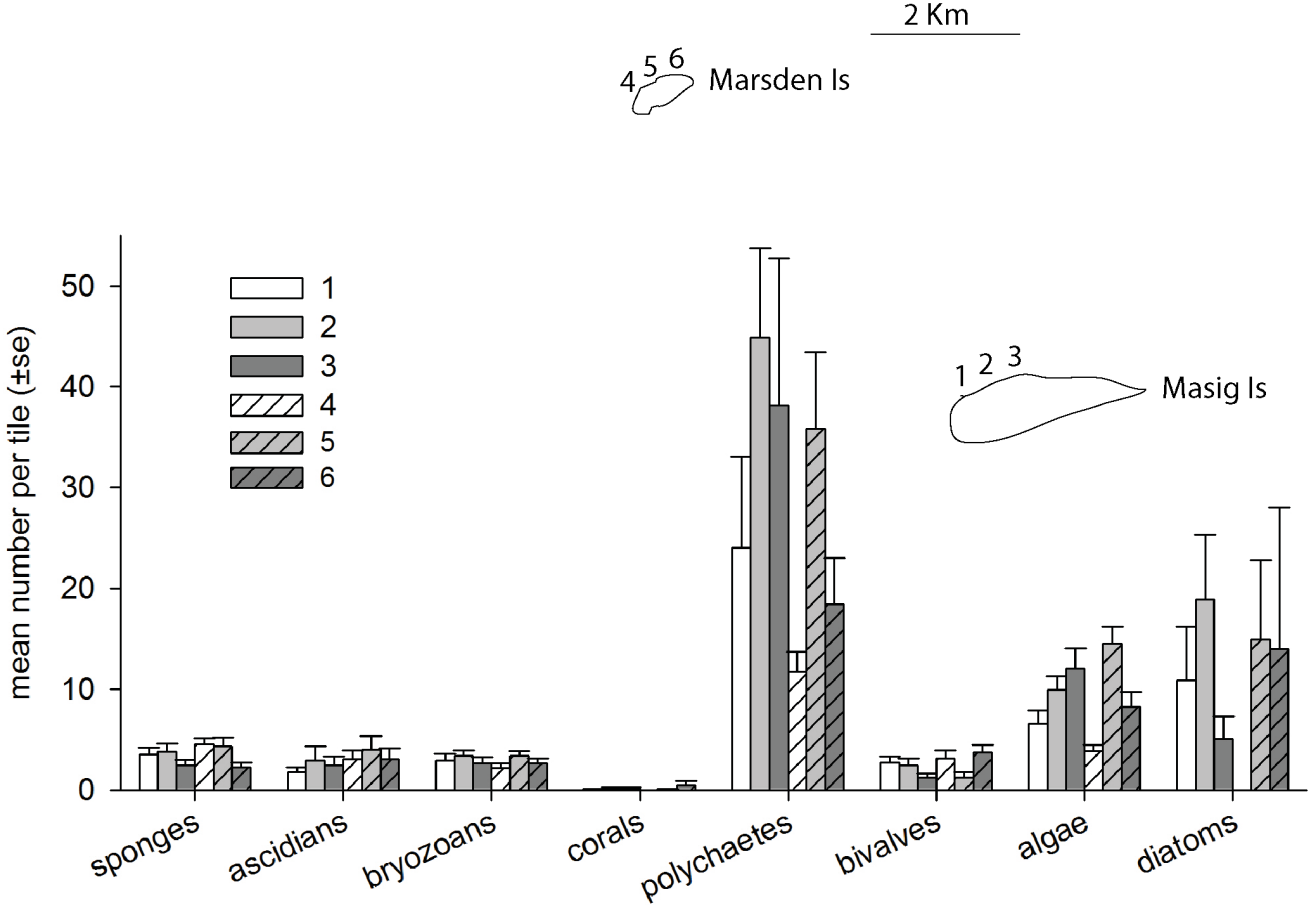
Mean abundance of each taxa, separated by locations, during the summer of the year 2 sampling period. Note locations 1 to 3 are Masig Is and 4 to 6 are Marsden Is. Refer to S1 Fig for the location of the islands within Torres Strait, Australia.

**Fig 3:**
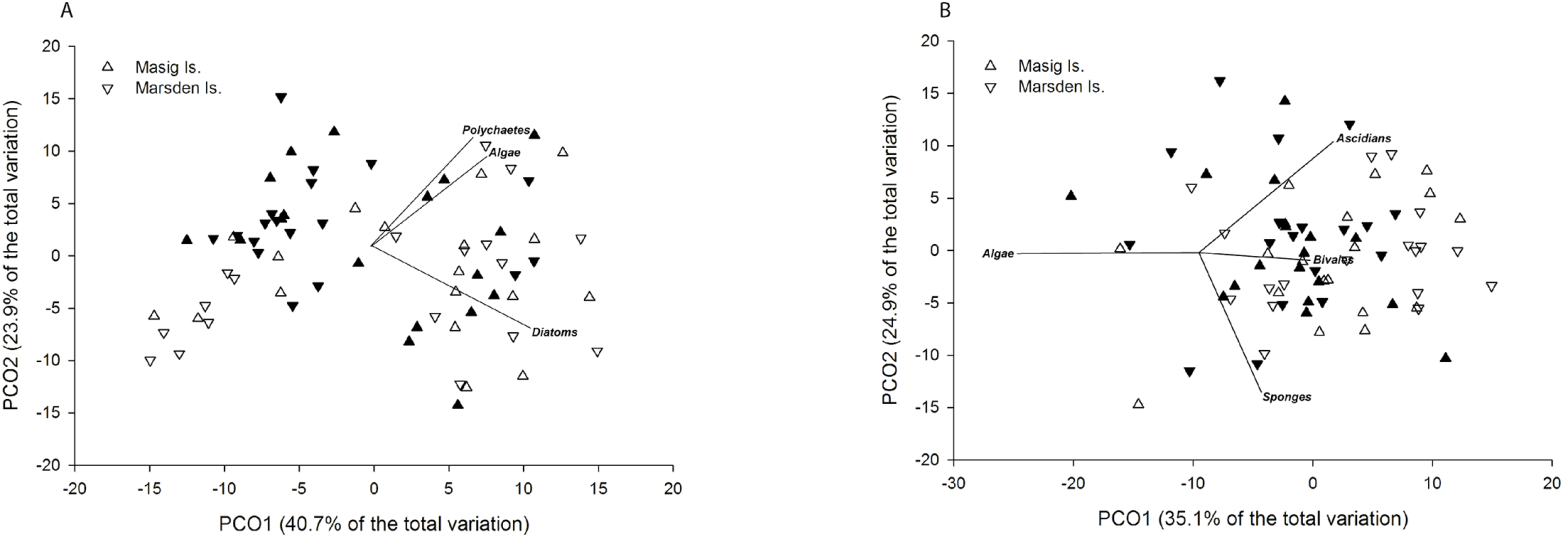
Principle coordinate analysis (PCoA) of invertebrate abundances (A) and assemblage structure, using percent cover data (B), at Masig and Marsden Islands. The five replicate tiles from each depth were pooled prior to constructing a Bray-Curtis similarity matrix. Taxa with a Spearman Rank correlation value greater than 0.6 have been overlaid on the plot as vectors. Summer and winter sampling periods are represented by open and closed symbols, respectively.

Recruitment abundances were similar at the highest spatial scale (e.g. between Islands) and across seasons; however, there was a significant effect of location and depth on recruit abundances (Table 1). Variation in recruitment abundance between locations was more pronounced for certain taxa including polychaetes, algae and diatoms, particularly during summer (Fig 2). This finding was also apparent in the PCoA, with the same groups contributing most to the discrimination (Fig 3A). The PCoA showed 64.6% of the variation explained in the first two axes, with no clear patterns separating recruitment between Islands or Seasons (Fig 3A). When examining assemblages using percent cover data, recruitment was remarkably similar across multiple spatial scales and between seasons, with PERMANOVA revealing depth to be the only significant source of variation (Table 2). This was further demonstrated with PCoA, with the ordination displaying no distinct separation between assemblages at the highest spatial scale (Fig 3B). Sixty percent of the total variation was explained in the first two factors, with algae, sponges, ascidians and bivalves contributing the most to the discrimination (Fig 3B).

**Table 1:** Results of the multivariate PERMANOVA for abundance data. Permutations were based on a Bray-Curtis similarity matrix generated from log(x+1) transformed data. Significant p-values (<0.05) in bold.

Source	df	Pseudo-F	p
Season	1	6.76	0.249
Island	1	0.41	1
Season x Island	1	0.59	0.59
**Location (Island)**	**4**	**2.44**	**0.005**
Site (Location)	12	1.06	0.382
Season x Location	4	1.04	0.438
Season x Site	12	0.94	0.65
**Depth (Site)**	**18**	**2.17**	**0.001**
Season x Depth	18	1.15	0.203

**Table 2:** Results of the multivariate PERMANOVA for percent cover data. Permutations were based on a Bray-Curtis similarity matrix generated from log(x+1) transformed data. Significant p-values (<0.05) in bold.

Source	df	Pseudo-F	p
Season	1	9.67	0.256
Island	1	0.34	0.904
Season x Island	1	0.61	0.616
Location (Island)	4	1.78	0.089
Site (Location)	12	1.4	0.142
Season x Location	4	1.32	0.273
Season x Site	12	1.04	0.438
**Depth (Site)**	**18**	**2.04**	**0.001**
Season x Depth	18	0.903	0.668

**Fig 4:**
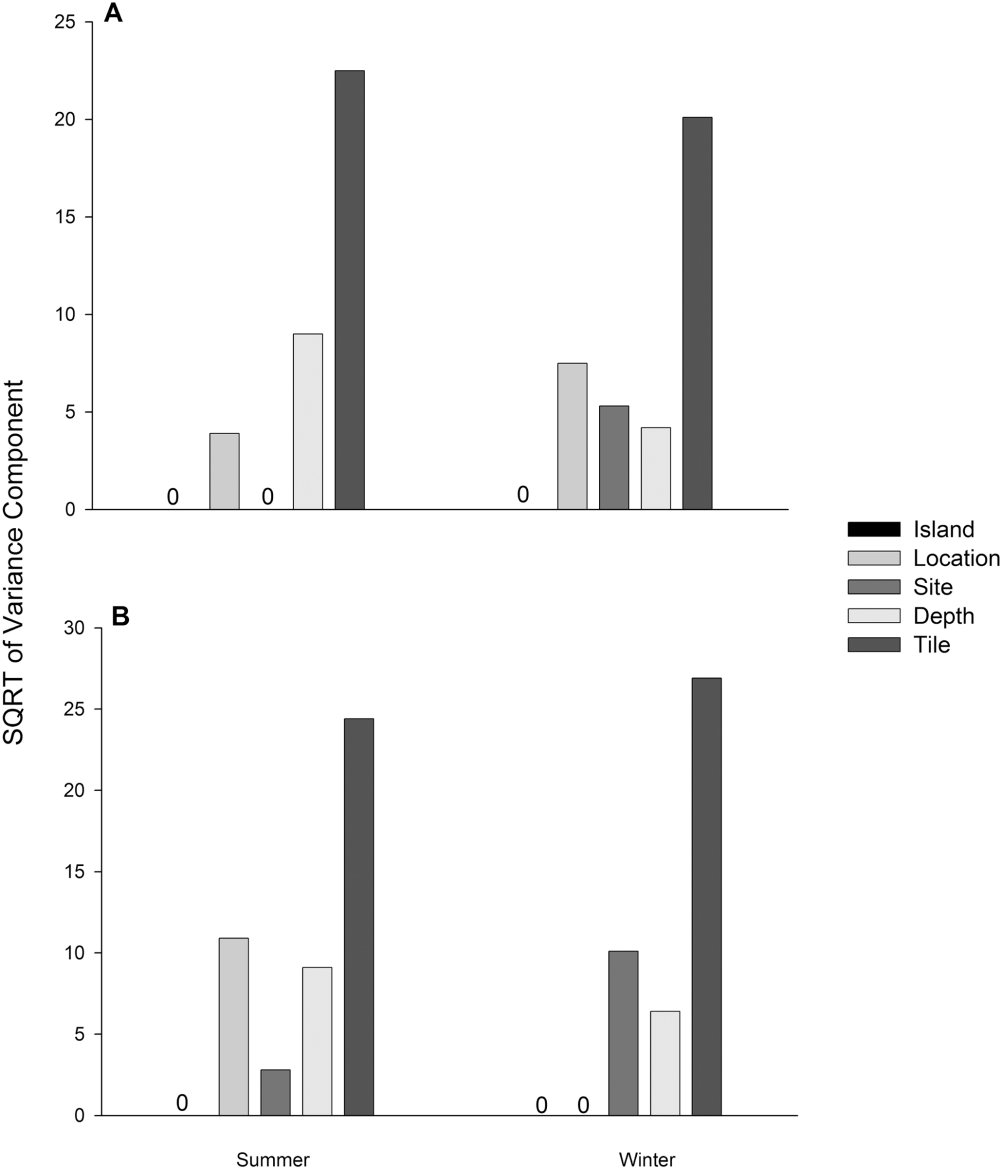
Variance components for (A) abundance and (B) percent cover for the Year 2 summer and winter. Note the different y-axis scale bars.

When recruitment cover was examined separately for each season, PERMANOVA revealed no significant differences at the higher spatial scales (e.g. islands, locations or sites) for summer or winter (Table 3). In fact, depth was the only significant factor, but only during the summer sampling period (Table 3). Similarly, recruitment abundances were not significantly different at the highest spatial scale for either season; there was a significant difference between locations during the summer only (Table 3). Examination of the pseudo-variance components from the PERMANOVA model revealed that the largest source of variation could consistently be attributed to the smallest spatial scale (i.e. between tiles, 1-m apart) for both recruitment abundance and percent cover (Fig 4A and 4B). The patterns of variation were inconsistent for both measures between the two seasons. For instance, Site contributed to the variation in abundances during winter, but not during summer (Fig 4A), and location contributed to the variation in percent cover during the summer, yet had no contribution during winter (Fig 4B). The only consistent source of variation between both measures and seasons (excluding tiles) was depth; however, it was higher for both during the summer (Fig 4A).

**Table 3:** Results of PERMANOVA tests to examine differences at both time points (i.e. summer & winter) separately. Permutations for abundance and percent cover were based on a Bray-Curtis similarity matrix generated from log(x+1) transformed data, while permutations for the individual taxa (using percent cover data) were based on a Euclidian distance matrix using untransformed data. Significant p-values (<0.01 to account for multiple tests) in bold. Is = Island, Lo = Location and Si = Site. Due to their very low abundances, corals were excluded from the individual PERMANOVA tests.

	Summer								Winter							
	Is		Lo (Is)		Si (Lo)		De (Si)		Is		Lo (Is)		Si (Lo)		De (Si)	
	F	p	F	p	F	p	F	p	F	p	F	p	F	p	F	p
Abundance	0.301	0.907	2.11	**0.016**	0.746	0.879	2.01	**0.001**	0.599	0.798	1.88	0.028	1.57	0.033	1.21	0.108
Percent cover	0.368	0.699	2.4	0.017	1.075	0.393	1.7	**0.002**	0.452	0.897	1.07	0.423	1.58	0.076	1.28	0.111
Algae	0.539	0.504	4.08	0.032	0.482	0.905	3.76	**0.001**	0.393	0.597	4.89	0.0164	0.7	0.74	2.28	**0.004**
Sponges	2.5 E2	0.901	1.39	0.282	0.694	0.739	2.11	**0.006**	3.14	0.097	0.323	0.849	1.52	0.204	0.924	0.553
Ascidians	1.3 E3	1	1.82	0.201	0.903	0.56	2.45	**0.002**	7.04	0.094	0.189	0.941	1.33	0.278	1.29	0.21
Bryozoans	2.64	0.196	0.148	0.973	1.76	0.132	0.846	0.649	0.424	0.497	1.28	0.328	1.21	0.343	1.36	0.155
Polychaetes	1.73	0.305	0.683	0.629	0.881	0.575	1.39	0.138	1.9 E2	1	2.06	0.131	1.08	0.432	0.918	0.560
Bivalves	0.558	0.599	8.14	**0.003**	0.565	0.842	1.15	0.314	0.357	0.899	1.40	0.286	1.95	0.087	1.07	0.393
Diatoms	0.432	0.901	3.33	0.031	1.79	0.093	0.689	0.849	1.22	0.394	0.909	0.479	2.09	0.060	0.667	0.878
Total significant		0		**2**		0		**5**		0		0		0		**1**

Individual PERMANOVA tests for each taxa revealed that depth was a significant source of variation during the summer for only three out of the seven taxa: algae, sponges and ascidians (Table 3). For instance, sponges covered a larger percentage of deep tiles, whereas ascidians covered a larger percentage of shallow tiles (Fig 1B). During the summer, there was also a significant difference in the cover of bivalves between locations (Table 3). Interestingly, the only significant source of variation in the winter was depth, but only for algae (Table 3).

**Fig 5:**
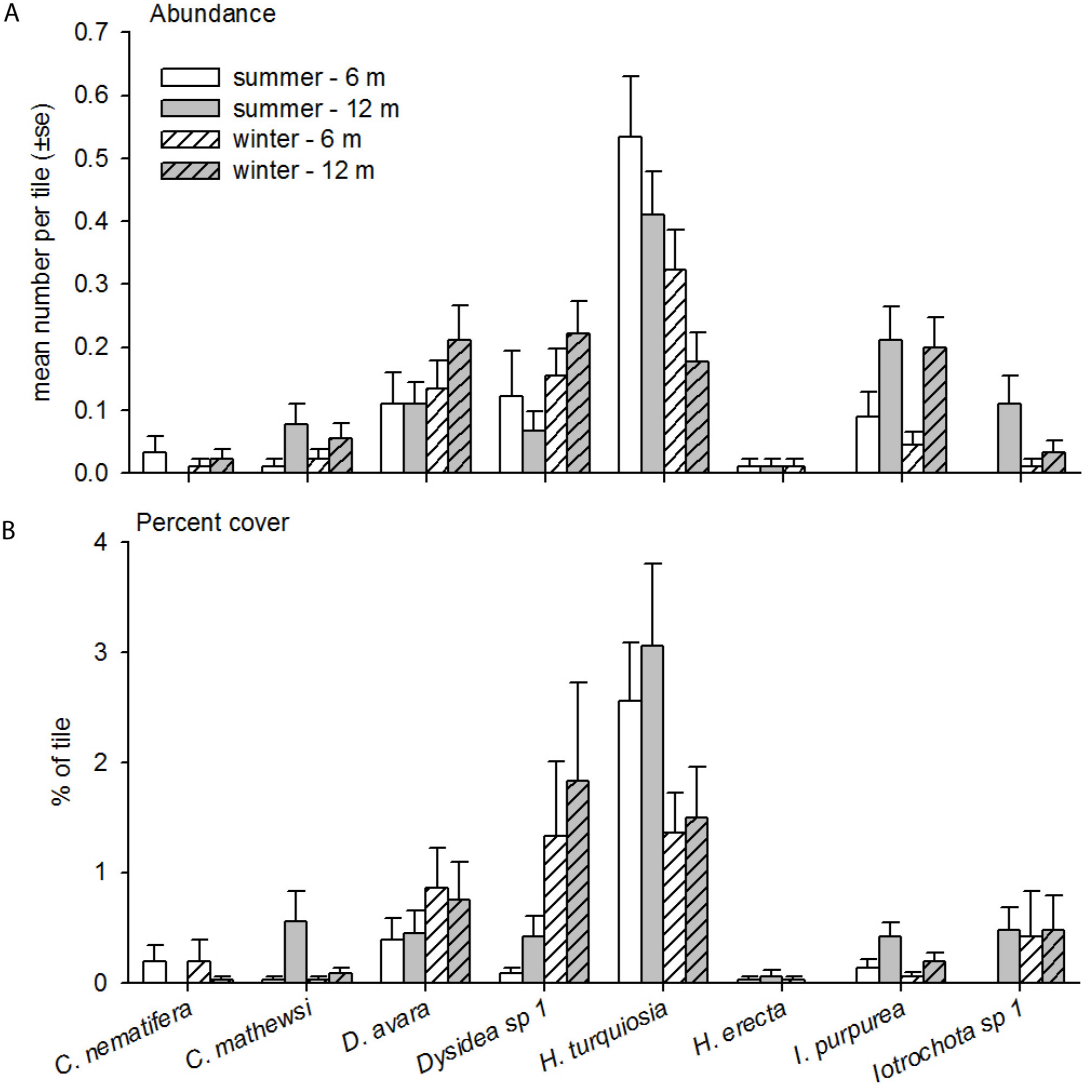
Mean abundance (A) and percent cover (B) of each sponge, positively identified to species level, for the year 2 sampling. Both seasons and depths depicted.

In total, eight different sponge species were positively identified over the course of the two-year study (S3 Fig). Species spanned six families and included: *Chalinula nematifera*, *Coscinoderma matthewsi*, *Dysidea avara*, *Dysidea* sp. 1 grey, *Haliclona turquiosia*, *Hyrtios erecta*, *Iotrochota purpurea* and *Iotrochota* sp. 1 green. Average recruitment abundances of all species were very low, with *H*. *turquiosia* recruitment being the highest (Fig 5A). Interestingly, *H*. *turquiosia* also occupied the highest percentage (overall mean = 2.12%) of the tiles, along with *Dysidea* sp. 1 grey (0.92%) (Fig 5B). *H*. *erecta* was the least abundant and occupied the smallest percentage (0.03%) of the tile out of all the sponges species identified (Fig 5A and 5B). Notably, higher recruitment of sponges like *Coscinoderma matthewsi* and Dysidea species at 12 m compared with 6 m agrees with the abundance patterns of adult sponges across depth [40](Fig 1B).

## Discussion

A notable finding of this study was that the highest levels of recruitment variation occurred at the lowest spatial scale examined, with recruitment varying more between experimental tiles 1 m apart than between sites, locations and islands. Recruitment variability of sessile benthic taxa, at small, within-habitat scales is a consistent finding in recruitment studies [38,39,41,42], despite the use of uniformly sized settlement tiles that provide a standardised habitat that limits recruitment variability associated with complex heterogeneous natural reef habitats [33,36]. Interpretations of small scale (i.e. highly localised) recruitment variability can be linked to a range of environmental processes, including competition for space or predation [41,43,44]. Physical processes including, boundary flow hydrodynamics and habitat surface topography can also play roles in recruitment variability at small spatial scales [45–47]. Tiles in this study had similar habitat topography; however, factors such as flow rates and light intensity were possibly different [45], which likely affected recruitment patterns.

The finding of recruitment heterogeneity among experimental tiles provides an important insight into the dynamics of recruitment. Heterogeneity at local scales, covering metres, suggests that local drivers (e.g. predation and competition) play a role in contributing to community assemblages at these spatial scales. Here, grazing (direct and incidental) from herbivorous fish can contribute to coral recruit mortality [14], which arguably translates to recruitment variability. The spatial scale of recruitment variability through predation/grazing may depend on the home range of herbivores. The two conspicuous groups of herbivores on coral reefs, fishes and urchins, show foraging patterns over a range of spatial scales [48,49] with both groups playing likely roles in contributing to recruitment variability over smaller within habitat scales[42], particularly to upper tile surfaces.

In the present study there was little evidence of recruitment to the upper surfaces of tiles. It is likely that the very low recruitment of organisms (e.g. algae) to the upper surfaces of tiles resulted from high grazing pressure and other post-settlement mortality [14]. Although not quantified, the upper surfaces of many tiles in this study possessed noticeable feeding scars. Similarly, nearly all recruits (98.8%) settled to the bottom of the tiles in a study done in the southern Persian Gulf [50]. In comparison, the underside tile surfaces, with clear signs of invertebrate and algal assemblages, potentially provided protection from larger grazers, with this pressure potentially being less important than other processes, including competition. In addition, light no doubt played an important role for coral and algal recruitment. Recruitment to under sides of tiles may also reflect recruitment of cryptic taxa to shaded habitats [42].

The small numbers of scleractinian coral recruits (<1 recruit per tile), to either the upper or bottom surfaces of tiles, was surprising, and is in contrast to other recruitment studies which demonstrate coral recruitment on both upper and under sides of settlement tiles and in higher numbers than observed in this study [16,17,27,29,31]. While patterns of scleractinian coral recruitment to individual experimental tiles can reflect nil, or very low numbers of recruits [17], average numbers of recruits in these comparative studies are conspicuously higher than scleractinian coral recruitment found in the present study, despite the study sites being located within a thriving coral reef community. For instance, coral recruitment on the GBR can range from 36 to 7000 recruits per m^2^ per year depending on the study and method employed (See Table 4, [51]). Incidental grazing from herbivores may be indicative of the low number of recruits, including corals, to the upper surfaces of tiles in this study, and the lower number of coral recruits underneath tiles may be a reflection of competition with other sessile invertebrate taxa [27]. Although not quantified as a part of this study, a relatively low abundance of crustose coralline algae (CCA) was observed on the underside of the tiles, potentially contributing to the low coral recruitment observed.

Polychaetes were by far the most abundant taxa observed, with abundances four times higher than any other taxa. However, this group occupied less than 5% of the tile surface. Polychaetes are known to recruit to dead coral in northern Australia [52] and are often found dominating recruitment of artificial structures [36,53]. This is an interesting finding given polychaetes are not a conspicuous group occurring on substrata in the immediate vicinity of experimental recruitment tiles. The immediate sessile reef community is predominantly comprised of cnidarians and sponges [34]. Nevertheless, polychaete diversity, and the apparent common occurrence of this group within coral reef micro habitats, is noted at Lizard Island, northern GBR [54]. That polychaetes, and to a lesser extent diatoms, dominate recruitment tiles may be a reflection of these groups excelling as colonisers rather than competitors. The dynamic between colonisers and competitors is routinely reported, particularly when recruitment surfaces represent bare space [36]. Therefore, tiles deployed for the six months in this study are likely to confer advantages to important colonising taxa such as polychaetes. However, the fact that polychaetes occupy a small area of space on tiles suggests less capacity for polychaetes as competitors, particularly when compared to taxa with noted encrusting habits or allopathic capacities such as ascidians, sponges and bryozoans resulting in overgrowth of poor spatial competitors [55–57].

Spatial recruitment variability was not evident beyond the smallest scales examined in this study (experimental tiles), suggesting that recruitment between islands and among locations within islands is less heterogeneous. While processes such as predation contribute to coral reef recruitment patterns at both small and large spatial scales, it is more likely that processes governing larval supply, and larval dispersal, are more uniform between islands or among locations within islands thereby contributing to less differentiation in overall recruitment. Despite the distance of several kilometres between islands, or among locations within islands, it is likely that there is enough localised dispersal and recruitment, limiting spatial heterogeneity among sessile groups. Larval dispersal for many coral reef sessile invertebrates can be highly endogenous, but with enough long-distance dispersal and recruitment to maintain population connectivity over regional scales [9,58].

This study also examined recruitment variability between shallow (6 m) and deep (12 m) sites. In this case, depth was found to be an important factor influencing recruitment during the summer, particularly for algae, sponges and ascidians. Sponge recruits were more prevalent and occupied a greater percentage of tiles at deeper sites, while ascidians on the other hand dominated shallow sites. While information of ascidian distribution patterns is unknown within Torres Strait, sponge distribution patterns are well documented [34,59]. The finding of higher sponge recruitment numbers at deeper sites (12 m) is consistent with adult distributions where sponges are most commonly observed between 12–15 m [34,59,60]. Interpreting processes that contribute to higher sponge abundance at deeper sites is complex. However, physical factors such as flow rates and light intensity would likely be different [59], which might affect recruitment patterns. Moreover, larval dispersal for some sponges can be driven by clear larval settlement behaviours that can cue larvae to settle in accordance to light and reef associated environmental/habitat cues [61–66]. Key environmental settlement cues suitable to sponges may be more commonly encountered at deeper sites and therefore may play important contributing roles to successful recruitment there.

## Conclusions

The finding that recruitment variability was highest at the smaller spatial scales examined in this study highlights the heterogeneity that occurs within habitats (i.e. at spatial scales of metres). While a range of both biotic and abiotic processes may contribute to patterns of recruitment in marine benthic community assemblages over small and regional scales, the higher variability within habitats suggests localised processes associated with competition and predation may play important roles in the heterogeneity of community assemblages on very fine scales. Torres Strait is situated at the northern boundary of the GBR with shallow reef habitats dominated by scleractinian corals. The very low numbers of coral recruits found in this study, and that it differs from other recruitment studies undertaken on the GBR, identifies a need for further work to bridge the complex temporal and spatial patterns of recruitment on coral reefs [51]. The low presence of organisms on the surface of tiles and encrusting organisms on the underside of tiles further highlights the potential role of both predation (through incidental grazing) and competition in defining the community assemblages. The documentation of non-coral sessile invertebrate recruitment patterns provides much-needed information on these groups within the northern GBR and more broadly coral reef systems. In addition, this study provides knowledge of key performance indicators related to coral community recruitment patterns that depict variability over time and space, which are valuable to how coral reefs are managed and conserved.

## Supporting Information

S1 Fig Map of the central Torres Strait, showing the two study Islands: Masig and Marsden (A). Diagram of the nested hierarchical study design (B).
(TIF)

S2 Fig Mean abundance (A) and percent cover (B) of each of the taxa from Masig and Marsden Is. over the two-year sampling period. Both seasons and depths depicted.
(TIF)

S3 Fig Mean abundance (A) and percent cover (B) of each for sponges, positively identified to species level, over the two year sampling. Both seasons and depths depicted.
(TIF)

S1 Table Abundance data. Raw abundance data for the year 2 sampling (May 2007 to May 2008).
(XLSX)

S2 Table Percent cover data. Raw percent cover data for the year 2 sampling (May 2007 to May 2008).
(XLSX)

S3 Table Sponge species data. Raw abundance and percent cover data for the sponge species identified in the year 2 sampling (May 2007 to May 2008).
(XLSX)
