# Supplementary figures and images for: Recruitment Variability of Coral Reef Sessile Communities of the Far North Great Barrier Reef
Source: PLoS One. 2016 Apr 6;11(4):e0153184. doi: 10.1371/journal.pone.0153184 (PMC4822782; doi:10.1371/journal.pone.0153184)

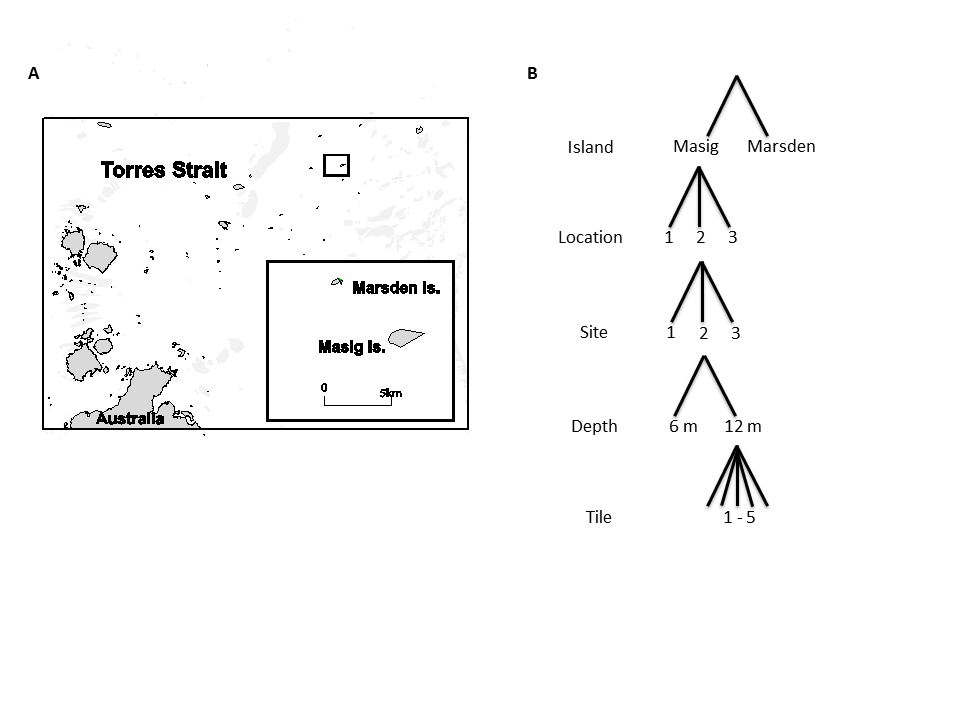

Supplement: S1 Fig — Map of the central Torres Strait, showing the two study Islands: Masig and Marsden (A). Diagram of the nested hierarchical study design (B). (TIF) [file pone.0153184.s001.tif]

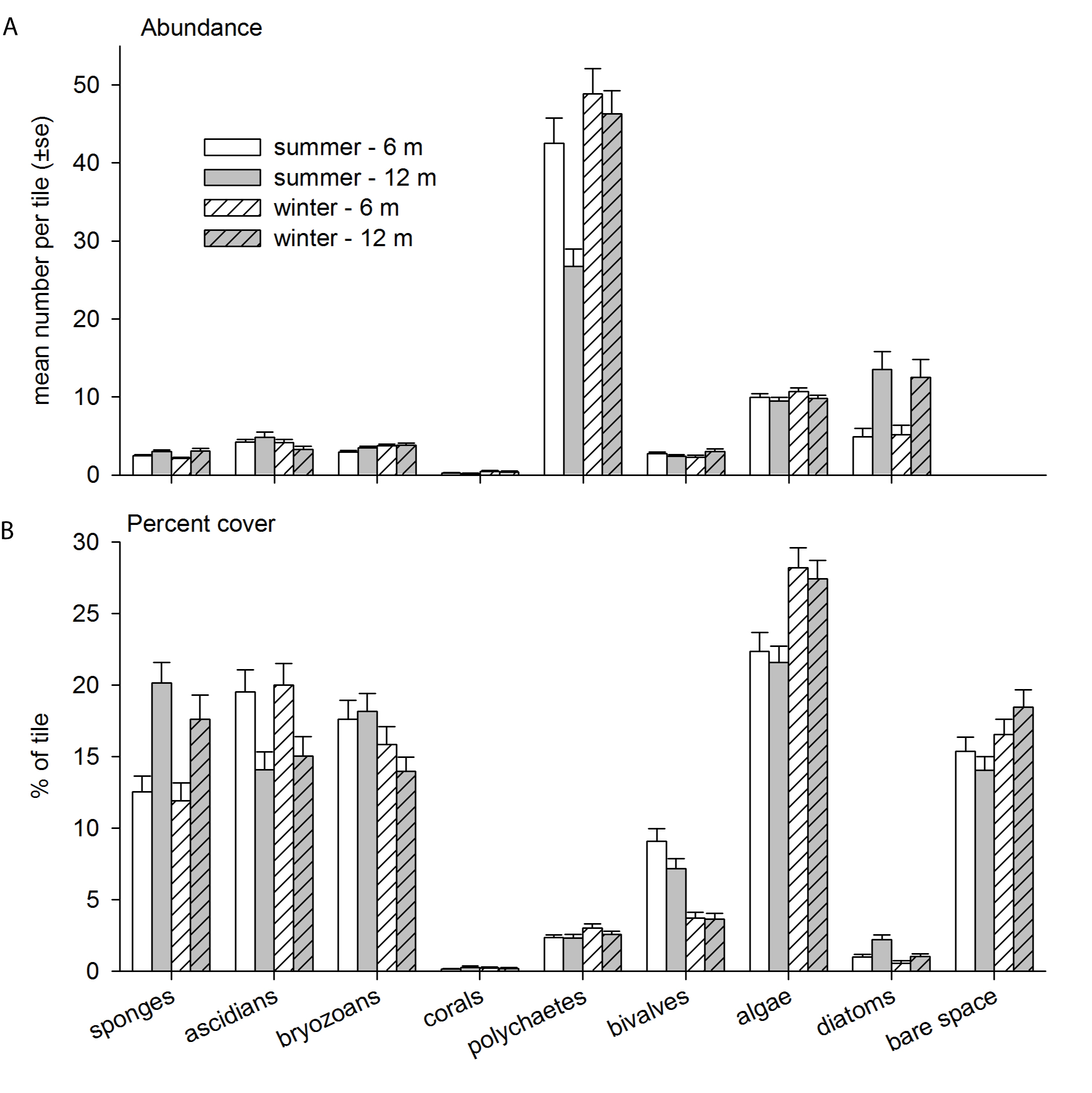

Supplement: S2 Fig — Both seasons and depths depicted. (TIF) [file pone.0153184.s002.tif]

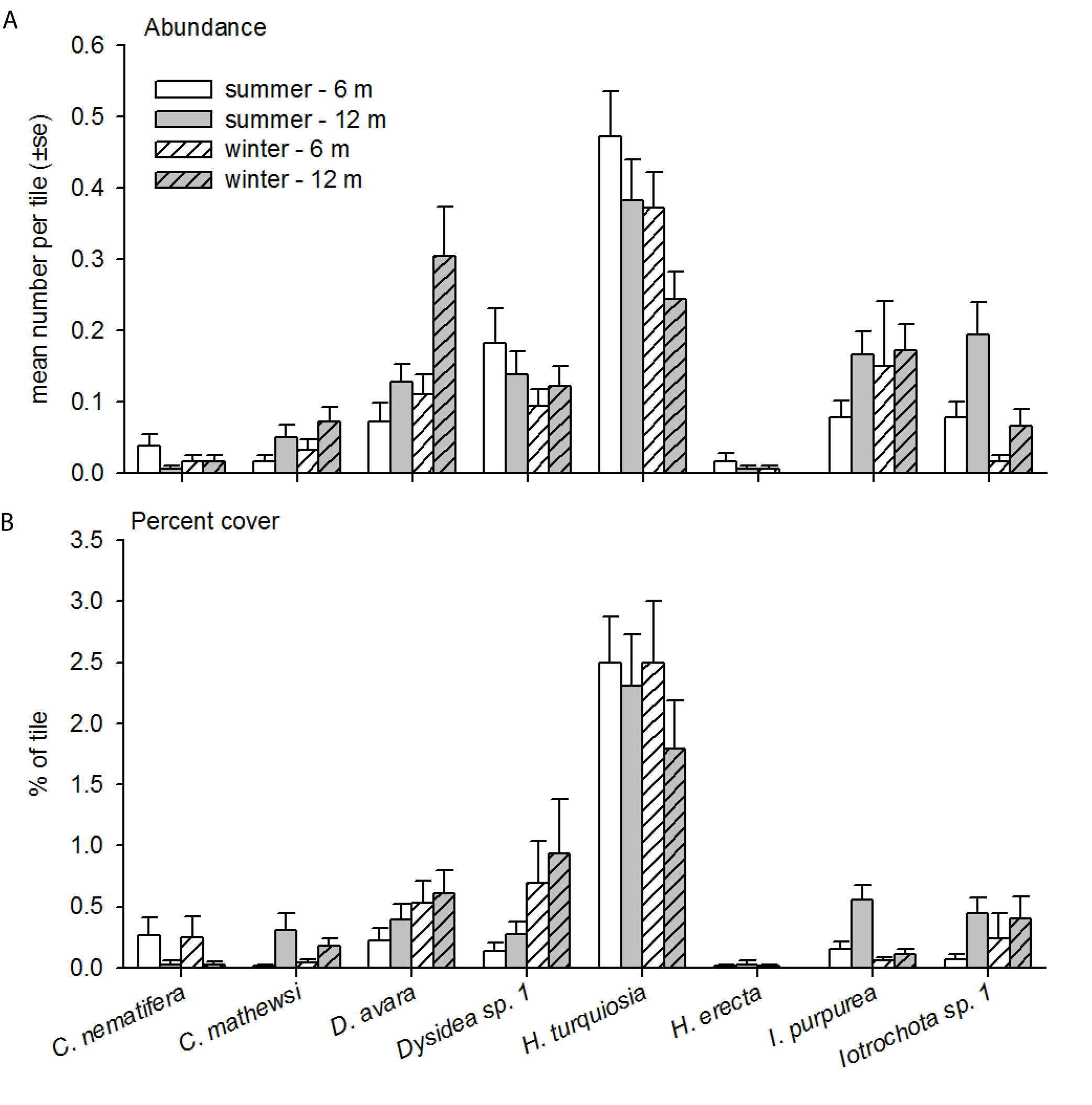

Supplement: S3 Fig — Both seasons and depths depicted. (TIF) [file pone.0153184.s003.tif]
